# Farmer experiences with virtual fencing: Adoption, adaptation, and farm system change in New Zealand pasture-based dairy systems

**DOI:** 10.3168/jdsc.2025-0983

**Published:** 2026-03-12

**Authors:** Callum R. Eastwood, Brian Dela Rue, Lucy Hall

**Affiliations:** 1 Research and Science, DairyNZ, Lincoln, New Zealand 7647; 2 Research and Science, DairyNZ, Hamilton, New Zealand 3240

## Abstract

Virtual fencing (VF) is a livestock management technology that uses global positioning systems–enabled collars and audio or electrical cues to geo-fence and herd animals. Virtual fencing is now in use on farms in several countries; however, there have been limited studies on the use of VF on commercial dairy farms and assessing the implications for how farm systems are managed. The aim of this study was to explore farmer experiences with VF use on pasture-based dairy farms in New Zealand. A qualitative research approach was used, with 60- to 90-min semistructured interviews conducted with 20 New Zealand dairy farmers (participants). Interviews were transcribed and analyzed using inductive open coding through Atlas.ti Web software (version 7.4.0-2024-04-04). Innovation appeal, improved job satisfaction, and reduced labor demands were dominant drivers for adoption. Adaptation of animals and people to the new system occurred within a few days or weeks following implementation of VF; however, more time was required for farm system adaptations, such as laneway modifications, additional water troughs, and removal of some permanent fencing. Post-adoption benefits, as perceived by participants, related to time savings, greater control of grazing and crop management, and herd management improvements. Although it was difficult for participants to place a financial return on their VF investment, almost all expressed an ongoing commitment to using the technology, indicating the presence of strong nonfinancial benefits. Findings from this research highlight the transformational potential of VF in grazed dairy systems, while underscoring the need for user-friendly interfaces, robust support systems, and strategies to support the early learning process and mitigate cognitive load.

## Highlights

• Virtual fencing enables flexible grazing and reduces labor requirements.
• Farmers report improved work-life balance and reduced stress.
• Adoption was driven by innovation and human factors more than by financial factors.
• Infrastructure changes (laneways, troughs) are critical for successful integration.

## Graphical Abstract

In this study, semistructured interviews were conducted with 20 New Zealand dairy farmers who were early users of a new virtual fencing technology. Drivers for adoption centered around the appeal of being involved in a new innovation, saving time on farm, and improving job satisfaction. Early implementation required adaptation for cows and people, along with a changed mindset about how to run the farm. Benefits seen by farmers included enhanced control of grazing, pasture utilization, and workplace attractiveness through reduced hours worked. The technology had implications such as high investment costs, potential need for infrastructure changes, changes to farm skills, and being locked into using the technology.

**Figure:**
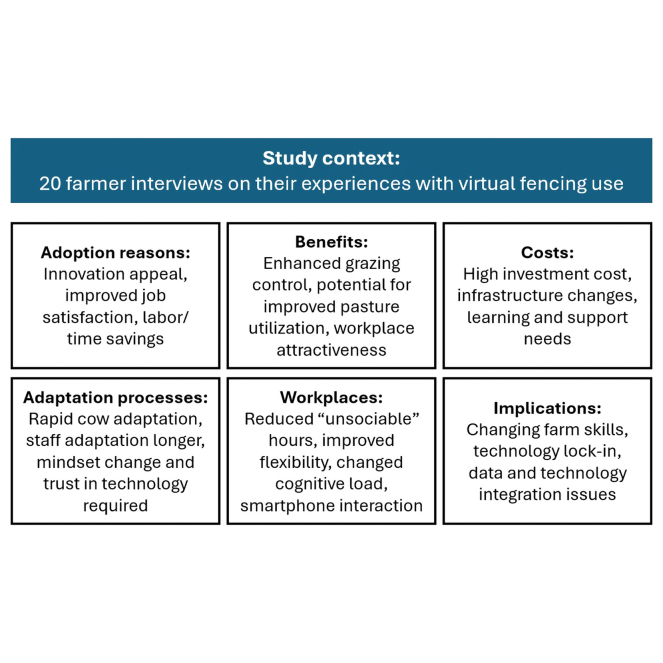

The use of on-animal sensors (wearable) technologies has increased in the past 5 years in New Zealand (**NZ**) dairy systems (Edwards et al., 2026). One of the more novel and potentially transformational wearable technologies is virtual fencing (**VF**). Virtual fencing technology has been in development for more than 20 years (Umstatter, 2011) and is now a commercial product for use with dairy and beef cows and goats (Langworthy et al., 2021; Eftang et al., 2022; Holohan et al., 2023; Schillings et al., 2024). In general, VF technology works through a collar-mounted device, worn around the neck of the animal. Devices provide location and behavior tracking via global positioning systems (**GPS**) and accelerometers, powered by a battery recharged through a solar panel unit. This ability to recharge sets VF devices apart from other accelerometer-based wearables that include a battery with a finite lifetime (O'Leary et al., 2020). The unique aspect of VF is the ability of the system to either “fence” or herd animals through a combination of sound, vibration, and pulse (shock) technology within the collar device, driven by software systems allowing users to set spatial (“virtual”) boundaries or remotely manage animal containment and movement.

New Zealand dairy farmers started using VF on their dairy farms around 2020, but information is limited about how the technology is being used and its potential effects on grazing management. Much published research relates to the efficacy of VF use for tasks such as containment and grazing management (Langworthy et al., 2021; Verdon et al., 2024), potential influence on animal welfare (Campbell et al., 2017; Verdon et al., 2021; Jeffus et al., 2025), and aspects of responsible innovation (Brier et al., 2020). Potential benefits of using VF in grazing systems include saved time through reduced need for herding and setting breaks; greater control over cow location and therefore improved ability to enact grazing plans; reduced need for erection and maintenance of physical fences, especially around waterways and for strip grazing; and individual animal management. Other potential benefits include enabling greater within-farm biodiversity through lowered fencing costs and more dynamic animal management, protection of environmentally sensitive areas such erosion-prone land and riparian areas, and temporary or seasonal access to previously unavailable areas or flood-prone pastures (Brier et al., 2020; Box et al., 2023; Murray et al., 2025). However, most of these benefits have only been theorized by researchers or promoted by technology suppliers and proponents based on anecdotal evidence. Empirical research-based evidence of use in practice is required to understand the practical implementation of VF. The aim of this study was to explore farmer experiences with VF use on pasture-based dairy farms in NZ. The insights will help guide farmers' use of this emerging technology, as well as inform rural professionals and researchers on the opportunities and challenges associated with VF use.

A qualitative, semistructured method was used to examine the use of VF on dairy farms. Semistructured interviewing, where researchers follow a question set but manage this through a conversation with the participant, is a widely used social research methodology (Thompson et al., 2019; Ritter et al., 2023). Twenty interviews were conducted in 2023 and 2024 by the authors, 10 each in the Canterbury and Waikato regions of NZ. Farmers interviewed were using a VF technology (Halter, Auckland, NZ) that was in an early innovation stage (Marmont et al., 2024) and had been using the technology for 1 to 3 years. Results presented in this paper should therefore be interpreted within this early-innovation context. The farmers interviewed operated businesses with herd sizes ranging from 210 to 3,500 cows, with flat to rolling topography; no irrigation to full-farm pivot irrigation; a range of adoption of other technologies; owner-operator family farms, sharemilkers and multifarm businesses; and block calving either in spring or autumn. Each interview had 5 sections: farm system description, investment rationale, use of technology for operational management, data and interface interaction, and workplace impacts. Question design was undertaken by the authors and pilot tested with 2 farmers using VF. Participants were identified using purposive sampling through professional networks and were selected to provide a range of farm systems, sizes and ownership structures. They were approached initially by phone with a follow-up email about the study, including a plain-language description and a consent form. Interviews were conducted until saturation of themes were noted by the researchers. No farmers refused to participate in the study.

Interviews were conducted at the participant's farm with 1 or 2 of the authors at each interview, which lasted 60 to 90 min. Interviews were voice-recorded, transcribed, and analyzed using a grounded theory orientation and using inductive content analysis methodology with ATLAS.ti Web software (version 7.4.0-2024-04-04; ATLAS.ti, 2024). Inductive qualitative analysis involves the thematic coding of text-based data, where codes are developed through the analysis. In this study, the research team iteratively coded sections of the interviews and then met to revise the codes, until a codebook was created. This codebook was then used to guide the remaining analysis. Finally, major themes were identified from the coding outcomes. Here, we present the results and discussion in a combined form for brevity. To add farmer voices, we cite specific interviewee quotes using the notation “**QX**,” indicating quote number, and interviewees are identified by a numbered farmer pseudonym (“Farmer X”).

Farmers interviewed cited a range of investment drivers, including being involved with innovative technology (see Q1), improving job satisfaction, and achieving greater control over grazing or other aspects of the farm system. Many interviewees had an innovative mindset, in which they were willing to try new technology in a β form, where the hardware and software were still in development and may experience issues. Workplace factors were important, with farmers looking to use the technology to save time on a range of tasks, particularly the early “unsociable” hours involved with herding cows to the milking parlor (Edwards et al., 2023). Reducing overall hours required to run the farm and reducing reliance on employed labor were important aspects for participants, aligning with those identified in other dairy technology research (Dela Rue et al., 2019; Yeo and Keske, 2024). Also mentioned was the goal of reducing stress and fatigue and increasing their enjoyment of farming and social sustainability, similar to adoption of other transformational technologies such as robotic milking (Hansen and Stræte, 2020). Financial aspects appeared to be less of a motivator for those interviewed, supporting the findings of other studies of precision dairy technologies (Blasch et al., 2022; Tey and Brindal, 2022).

Technology is something that we've always grasped because we just want to be the first to try everything, because it keeps the job exciting for me and everybody else. (Q1, Farmer 5)

The early implementation phase on the study farms involved adaptation of the farm system to fit the technology, adaptation of animal behavior, and adaptation for the people working on the study farms. This phase was relatively rapid, with farmers noting that it only took cows a few days to 2 weeks to adapt to the VF system. Virtual fencing studies have shown similar timeframes for animal learning (Verdon et al., 2021). In terms of adaptation for people on farm, where people were using VF daily, it was only a couple of weeks before they became comfortable with the primary functionality, but it took longer for many to exploit the technology more fully (Q2). There was a process involved in getting used to the app as well as trusting the VF system (Q3).

We're still learning. We're still trying to develop how we run the farm with [VF]. But that was the big thing that they told us, when we enquired about it, was, “You will not farm the way that you always have.” You have to be willing to change your system and run it the way that's going to work best to utilize the technology or else you're not going to get the benefit out of it at such a high cost. (Q2, Farmer 9)

The first time you use it, you know, you're sort of checking the cows, are they staying on the fence? Like in the zone? (Q3, Farmer 6)

Some of the adaptation related to changes in mindset around farm management or more technical aspects such as learning how long a herd takes to walk to the parlor using VF, and changing practices around closing physical gates along the laneway between herds (Q4). Farmers learned that they had to have all gates that cows would pass in a laneway during herding closed, except the one where the cows are destined to go. This was because it was not guaranteed that the VF system would prevent cows going into gateways and then being stuck on the wrong side of the fence as the herd moved along the laneway. In conventional systems, gate closing would usually be done by a person who is herding the cows to the parlor, but with VF this needed to be undertaken before virtually “releasing” the herd from the paddock. When multiple herds used the same laneway, on some farms employees had to drive to open or close gates between milkings, negating a small portion of the time-saving captured through virtual herding. For this reason, and to avoid issues around wanting to use the same laneway for 2 different herds, interviewees noted that having multiple races leading to and from the dairy is especially useful for farms with VF (Q5). Farmers noted the need to adapt their farm systems to get the most out of the investment. These insights support other studies that identified farm systems adaptation required during the early implementation phase with precision farming technologies, particularly operational practice changes required to achieve value from technology (Eastwood et al., 2012; Hansen, 2015).

Before herds come in, we'll always do a lap to make sure gateways are closed so we know they're not going to go the wrong way and then as herds come into the shed is when we're setting up gateways. (Q4, Farmer 2)

I'd definitely improve my laneways and yard at the shed. So, with [VF] ideally, I can have one herd milking and the other one should be able to walk in behind from a different angle, but I can't do that kind of thing [because of current laneway layout]. They've got to be gone [first milking herd], so I've got to time it really well with [VF]. (Q5, Farmer 14)

A major area for adaptation was farm infrastructure changes, involving changes to paddock layouts and gateways, laneways, and water access (troughs). Several interviewees had removed some permanent fencing to create larger paddocks with the aim of leveraging VF capability and increasing grazing management flexibility. However, certain functionality of the VF technology meant that there were some limits to reducing fencing, and farmers also noted the need to balance management of uncollared stock such as calves and bulls. Sharemilkers (a form of share farming in NZ) were less likely to remove fences, as they might need to replace them when they left the farm. Investment in more water troughs was vital for farmers to use VF effectively, as each virtually fenced grazing area needed to have access to drinking water for cows. In a few cases laneways were also widened to make herding cows easier. The interviews highlighted that infrastructure changes need to be a key consideration for farmers contemplating VF investment, and that the cost of such changes should be included in investment business cases. Add-on or unforeseen costs have also been identified in other studies of precision farming, such as in robotic milking investment (Eastwood and Renwick, 2020).

Learning associated with new technologies and practices can be a challenge, and training is critical to successful implementation (Brennan et al., 2025; Ehlert and Brennan, 2025). Farmers noted that the VF company provided training and support, particularly pre-installation and in the initial period after installation. This training came in the form of one-to-one support, online chat groups, field days, and sharing videos with users talking about their use of VF. Training and support provided by different dairy technology companies has previously been shown to vary widely (Eastwood et al., 2012); therefore this aspect is an important one for farmers to consider when making VF investments. Given it was an early-stage innovation, most farmers interviewed felt they had a good connection to company support, often able to talk directly to software developers or the company leadership team. Responsiveness of customer support was identified by many farmers as a key feature in their implementation experience. Some farmers expressed tension around early performance of the technology and use of a developing technology, but there was general acknowledgment that issues were usually addressed (Q6). Farmers said their feedback was also incorporated into further development of the technology, highlighting a benefit of being involved in early-stage technologies as a form of collaborative design between technology developers and farmers (Ingram et al., 2022; Klerkx and Villalobos, 2024).

We knew that signing up, that there would be issues with the technology that would need to be rectified. And I suppose the positive has been that you make your complaint and the communication about how that's going on. If you've got an idea and you put it forward, even on the app or whatever, there's normally a reply within the day. If it's something urgent, it's within 20 min, you get to realize that things do get changed. (Q6, Farmer 9)

Interviewees identified the potential for improving grazing management and pasture utilization through VF use, for example from greater control over grazing, where break sizes could easily be created using square meters per cow adjusted for herd size and desired allocation. Different topography could also be accounted for more easily than with physical fencing. The ease of creating multiple breaks per day, without the time required to erect temporary physical fences, meant that small adjustments in allocation could be made, maintaining grazing pressure and reducing wastage from cows walking over fresh grass (Q7). The ability to create a virtual “back fence” was another benefit for keeping cows off previously grazed areas to prevent additional soil damage and to allow pasture regrowth (Farrell et al., 2024).

I set up 3 breaks for the young herd even though they're on once a day . . . not something you'd do with reel and standards. (Q7, Farmer 15)

Questions were raised by some interviewees as to the optimal use of VF for grazing management, as there was potential to spend more time making multiple breaks without achieving additional benefit. One farmer identified a risk that uncertainty was introduced into the herd with too many fence movements or poor virtual fence layouts. Overcomplicating grazing management, just because VF technology makes it possible, could lead to poor grazing outcomes, additional costs from extra troughs, or wasted effort. There is potential for improved pasture utilization and farm profitability from regular pasture mass measurement and more accurate allocation (Beukes et al., 2019; Neal and Roche, 2019). However, recent research has raised questions around the benefits in terms of animal and pasture performance from providing multiple grazing breaks per day (Farrell et al., 2024). One farmer noted that precise area allocation via VF is meaningless if the underlying pasture data are inaccurate. Some farmers identified benefits in grazing crops, especially because staff no longer needed to erect temporary fences through dense crops. However, it was also highlighted that for some forage crops precise allocation was required due to acidosis risk, and error in the GPS location for cows or device malfunction could lead to overallocation and animal health issues. Conversely, VF could provide the ability to reduce animal and farm system risks by enabling more precise management of crops, for example making it easier to have short crop grazing periods before moving cows back onto pasture.

In terms of its effects on cows, some farmers noted benefits around reduced lameness from reducing pressure during herding, as cows could walk at their own speed. Benefits around calving were also cited, where recently calved cows could have their collar “paused,” allowing them to move away from the herd and onto fresh pasture. At the time of the interviews, the VF system included a function to undertake in-paddock drafting of cows; that is, moving individuals or groups away from the main herd. Farmers using this function noted it required significant time to train cows to respond and move away from the rest of the herd. The VF technology has had continual evolution since the interviews were conducted, and the in-paddock drafting function is no longer available at the time of writing. Although the VF system also provided farmers with information for estrus detection, we did not explore that use in this analysis, as it would require a more detailed quantitative analysis (Edwards et al., 2026).

A main investment driver for VF was to improve the farm workplace through reduced hours worked, saved labor costs, and automating some less desirable tasks (e.g., herding cows to the parlor early in the morning, erecting temporary fencing for pasture or crops). Perceptions of time savings achieved varied among the farmers interviewed; some said they had saved sufficient time on farm to reduce a full (or part) employee, whereas others found more value from reducing undesirable tasks and improving attractiveness (Q8).

One part [of the reason for using VF] is the innovation side of it, and the other part was how can we make this farm nicer for people, and keep attracting better people, and retain your top ones as well. (Q8, Farmer 7)

The reductions in time spent on tasks around herding and break fencing were particularly beneficial on small farms with owner-operators, where it offered the potential to run the farm solo with only the need for a relief milker (Q9). In some cases, this meant the farmer could reduce the total hours required to run the farm or reduce aspects of physical work, allowing them to keep older staff members on the team. In many cases the overall weekly time savings were small, as time was reallocated to other tasks, but even small changes around the start or the workday or reduction in undesirable tasks had value for farm teams. Virtual fencing technology also provided some farmers with smaller herds with reassurance that they could temporarily run their farm solo if their employee quit. For owner-operators, and in some cases managers of larger farms, the technology provided them with more confidence to get off farm for periods during the day or for holidays. One farmer was able to take off-farm employment while staying connected to daily farm management via the VF app. A farm manager noted that VF use had reduced time spent informing absentee farm owners, as those owners could access the app to view the farm status.

If you're a 250- [to] 300-cow farm, and you want to drop your labor unit, just make life a hell of a lot simpler and have a relief milker, well, probably quite useful. (Q9, Farmer 11)

Farmers had a range of reflections on the interaction between farm staff and the VF system. Some noted that the implementation of VF and consequent changes to job tasks and roles could cause stress or disruption in the team, especially in the early learning period. Smartphones and associated apps are key tools in digital agriculture (Kenny and Regan, 2021), and, although almost all of the staff in this study had smartphones, there were a range of implications. The adoption of VF on some of the participant farms meant employees who were not confident using a smartphone, or who had an older smartphone that could not run the VF app, were effectively not able to engage with the new digital infrastructure. This exclusion, or the demands of learning a new app-based farming approach and fear of doing something wrong in the app, created stress among staff on some farms. However, in some cases, positive employee engagement with VF in the longer term was noted despite hesitancy and stress during the initial adaptation period (Q10).

One of our staff is an older guy who has been with us for about 8 years and not really into technology much, which sounds kind of strange considering the amount of technology we have, but he does adopt it and use it but he's not really into it. So, he was a bit stressed going into the VF process . . . we bought him a smartphone and got him all set up. Within a week of that he's like, “I'm never going to work on another farm.” (Q10, Farmer 8)

The use of precision technologies has been associated with changes in skills and roles in agriculture (Ingram and Maye, 2020; Schröder et al., 2022). Farmers in this study identified a range of implications for employees and dairy workplaces. One farmer felt that the technology could be used in lieu of a highly trained employee, and that with the VF system they could achieve the same outcomes with a less skilled team. Another felt that the spatial nature of the app would make it easier for people who are new to the farm, as they could visually see what was happening with herd management, grazing, and individual cows. In some cases, having VF “democratized” the farming process, as all employees could have access to the app and have greater interaction with farm activities and potentially make operational decisions themselves. In other cases, defined roles had been created around use of the technology, with a “go-to” person or technology champion who was most skilled with its use and would make most of the actions in the VF system. Several of the farmers noted that technologies such as VF had the potential to lead to changes to the skill base of farmers and employees, particularly around grazing management, animal husbandry, and the detection of estrus and animal health issues. Some felt that this was a natural transition in skills required for modern farming; however, others felt it led to employees becoming “lazier” or less engaged with the herd or the farm. Potential issues were also cited with employees moving from VF farms to non-VF farms and not having sufficient fundamental skills without technology supporting them. For some, it was changing what it means to be “farming.” A farmer noted that sometimes they wondered “Have I really farmed?” when they were creating grazing breaks from their app. Issues around the effects of digital technologies on farming practices and skills have also been highlighted in recent research (Butler and Holloway, 2016; Eastwood et al., 2023).

The effects of VF and its associated data were a double-edged sword for farm teams in terms of workplace stress and cognitive load (Q11). Constant interaction with the app was mentioned by some of those interviewed, and this could represent a positive or negative interaction (Q12). On the positive side, farmers appreciated the ability to carry out remote actions such as changing virtual fence boundaries, or releasing the herd for milking, from home (or even from bed). This added flexibility into their daily schedule and could mean additional time to sleep or be with family. Increased flexibility in dairy workplaces has been identified as one component for improving workplace attractiveness (Hogan et al., 2024). Additionally, some farmers noted that the option to view the current location of all cows at any point of the day or night via the individual GPS locations was reassuring, especially if they were away from the farm or if a severe weather event was occurring. One farmer said that they liked being able to shift the cows from home on a very wet night to avoid pugging damage. The ability to manage or assess the herd remotely via the app also gave some farmers confidence to get off farm for other tasks or to take time off. The inability of many farmers to get time off farm has been shown to affect well-being and job satisfaction (Hansen, 2022; Knook et al., 2024). On the negative side, the “always connected” nature of VF led some farmers and employees to feel they could not switch off. In “traditional” grazed systems, the workday effectively ends when the herd is put back in the paddock after the evening milking, allowing farmers a chance to switch off mentally. This constant connection to the farm workplace is similar to that seen in robotic milking systems (Hansen et al., 2020; Eastwood et al., 2022).

The cognitive load probably got worse. That's something I found surprising at the start. I was like, okay, I expected my life to get way easier. My life didn't get much easier. The [farm employee's] did. My life stayed very similar. But once I started offloading a bit to my [second-in-charge] in terms of running the actual cows—I still do all the breaks, but the rest I don't really touch anymore. So, it took a while to get to that point where I was willing to give up that control. But I'm happy now. (Q11, Farmer 17)

The biggest problem that I have with it—and my wife will tell you—is that you're connected all the time. That's the biggest problem. Whether we are just simply going to lunch, have dinner. It's only like, “Shit, I haven't done this.” I can do it right there, whereas before you would have had dinner and then walked out and done the fence. (Q12, Farmer 18)

In terms of benefits observed by farmers, almost all respondents stated a wish to keep using VF. The benefits can be categorized as financial and nonfinancial, with farmers often struggling to determine exact time savings, specific financial benefits, or a quantified return on investment. Nonfinancial benefits cited included many areas already discussed around reduced hours worked, greater feeling of control, and workplace attractiveness. There were unexpected benefits such as creating efficiencies through larger paddocks (Q13).

Because our paddocks are bigger, we negotiate a better deal with contractors, because they can get in and get out faster. The fertilizer spreaders [contractors] love it. (Q13, Farmer 19)

In terms of barriers and issues, ongoing subscription costs were the major factor, with one farmer noting, “If I won the lottery, I would have it on all my farms.” This cost was weighed against the nonfinancial factors such as work-life balance and staff happiness, as well as against the current milk price and profitability. Farmers noted issues common with precision technologies, such as technology interoperability and the ability to access and share the data from the VF system (Wolfert et al., 2017; Bahlo et al., 2019). Another issue was technological lock-in, where farm system adaptation and infrastructure changes could make it difficult to revert to farming without VF, similar to technological lock-in explored in other studies (Carolan, 2020; Eastwood et al., 2023). These issues need to be highlighted to farmers and the broader sector to ensure farmer awareness of potential farming change trajectories.

In summary, participants identified motivations for VF adoption that were often nonfinancial or difficult to accurately quantify. Innovation appeal, improved job satisfaction, and reduced labor demands were dominant drivers, whereas profitability was rarely cited as a primary factor. Overall, participants felt the use of VF technology was positive for their farming practices, particularly control over more intricate grazing practices and herd management. Study participants highlighted benefits such as making farm tasks easier, new decision-making data streams, and increased visibility over herd management practices. In terms of adaptation, most interviewees said cows acclimated to VF within 2 weeks of use, and staff adapted to VF functionality over a similar timeframe. However, VF integration potentially required changes in farm infrastructure, including laneway modifications, additional water troughs, and removal of some permanent fencing. These adjustments introduced unforeseen costs, underscoring the need to incorporate infrastructure investment into business cases for VF adoption. Other challenges included potential increases in cognitive load, greater digital dependency, and stress during the initial learning phase.

Together, the themes identified in this study help inform conceptual understanding of the adoption process of early-stage innovations. Although adoption of such technologies is often framed in economic or productivity terms (Yeo and Keske, 2024), insights from the farmers interviewed show wider social and farm system contexts. Adoption and implementation on these farms were dynamic, iterative, and nonlinear processes, supporting findings from similar studies (Montes de Oca Munguia et al., 2021; Langer et al., 2024). Themes included initial motivations (e.g., reduced labor, improved job satisfaction, innovation appeal), implementation processes (e.g., adaptation of cows, people, and infrastructure), burdens and enablers (e.g., digital dependency, cognitive load, interface usability), and anticipated benefits (e.g., grazing management, workplace design). Farmers were actively sense-making (Savolainen, 1993) to manage the tension between the implementation and adaptation of an evolving technology with anticipated system-level gains. Responses of those interviewed were largely positive regarding VF investment. It should be noted that such early users of technology often exhibit higher enthusiasm, risk tolerance, and motivation to overcome technology limitations (Pedersen et al., 2001). The positive narratives may also reflect a justification process, in which early adopters emphasize successful aspects to legitimize their decision to adopt an emerging and relatively unproven tool (Eastwood et al., 2012).

Findings suggest that VF adoption decisions should account for hidden infrastructure costs, training requirements, smartphone proficiency of the farm team, and workplace dynamics alongside anticipated labor savings. Technology developers and advisors should prioritize software interfaces that enable engagement by farm staff with a range of skills and personalities. Also required are training and extension approaches that support the early learning process and mitigate cognitive load and digital dependency stress among users. As use of VF becomes more widespread, there is a role for public or industry research and extension organizations to explore and foster innovative use of VF functionality to enable transformative opportunities for the dairy sector. Future research with a sufficient sample size should quantify economic trade-offs between labor reduction and adaptation costs, and likelihood and extent of productivity gains from improved grazing management, as well as exploring long-term effects on farm profitability, employee well-being, and farm system resilience.
